# To Do or Not to Do: The cerebellum and neocortex contribute to predicting sequences of social intentions

**DOI:** 10.3758/s13415-023-01071-x

**Published:** 2023-02-14

**Authors:** Naem Haihambo, Qianying Ma, Kris Baetens, Min Pu, Natacha Deroost, Chris Baeken, Frank van Overwalle

**Affiliations:** 1 Department of Psychology and Center for Neuroscience, Vrije Universiteit Brussel, Pleinlaan 2, B - 1050 Brussels, Belgium; 2 Department of Head and Skin (UZGent), Ghent Experimental Psychiatry (GHEP) Lab, Ghent University, Ghent, Belgium; 3 Department of Psychiatry, University Hospital (UZBrussel), Brussels, Belgium; 4 Department of Electrical Engineering, Eindhoven University of Technology, Eindhoven, the Netherlands

**Keywords:** Posterior cerebellum, Intentions, Social prediction, Social action sequencing, Cerebellar lobule IX

## Abstract

Humans read the minds of others to predict their actions and efficiently navigate social environments, a capacity called mentalizing. Accumulating evidence suggests that the cerebellum, especially Crus 1 and 2, and lobule IX are involved in identifying the sequence of others’ actions. In the current study, we investigated the neural correlates that underly predicting others’ intentions and how this plays out in the sequence of their actions. We developed a novel intention prediction task, which required participants to put protagonists’ behaviors in the correct chronological order based on the protagonists’ honest or deceitful intentions (i.e., inducing true or false beliefs in others). We found robust activation of cerebellar lobule IX and key mentalizing areas in the neocortex when participants ordered protagonists’ intentional behaviors compared with not ordering behaviors or to ordering object scenarios. Unlike a previous task that involved prediction based on personality traits that recruited cerebellar Crus 1 and 2, and lobule IX (Haihambo et al., 2021), the present task recruited only the cerebellar lobule IX. These results suggest that cerebellar lobule IX may be generally involved in social action sequence prediction, and that different areas of the cerebellum are specialized for distinct mentalizing functions.

## Introduction

Reading peoples intentions towards ourselves and others is a fundamental skill, one which we engage in countless times a day. For example, if we notice that a cashier gives another customer in front of us the wrong change, our assumption of whether they did this intentionally allows us to predict their actions towards ourselves and others in future. This process, which includes reading mental states, such as personality traits, beliefs, and intentions is termed mentalizing (Molenberghs et al., 2016; Schurz et al., 2014; Van Overwalle & Baetens, 2009).

An important consequence of mentalizing is that it allows us to predict, anticipate and plan subsequent behaviors (Frith & Frith, 2006; Molinari & Masciullo, 2019), allowing for smooth and efficient current and future social interactions. Take again, the example about the cashier above: if we assume the cashier gave the wrong change on purpose, we might further assume that they cannot be trusted to tell the truth if we confront them about it. Specific brain regions, such as the medial prefrontal cortex (mPFC), temporoparietal junction (TPJ), and precuneus/posterior cingulate cortex (PPC), have been associated with mentalizing and have collectively been termed the “mentalizing network” (Schurz et al., 2014; Van Overwalle, 2009), which takes up a major part of the default mode network (Andrews-Hanna, Smallwood, & Spreng, 2014b).

Along with these cortical areas, the posterior cerebellum—specifically Crus 1 and 2, as well as lobule IX—has been identified as being involved in social mentalizing (Pisotta & Molinari, 2014; Van Overwalle et al., 2014; Van Overwalle, Van de Steen, et al., 2020c). What is the function of the cerebellum in mentalizing? According to the traditional view of the cerebellum in motor processing, the cerebellum receives information about movements from the cerebrum, checks the match or mismatch with movement templates encoded in internal models, and “if a match is obtained, then it is assumed that the next wave of incoming sensory data can be predicted from the stored template.…This forward-looking cerebellar function enables the central nervous system to plan ahead” (Molinari et al., 2009, p. 399). In an evolutionary extension of this traditional function, the “sequencing hypothesis” (Leggio & Molinari, 2015) argued that a major function of the cerebellum is to identify and predict repetitive patterns of temporal sequences in the motor and nonmotor domain (e.g., executive and social cognition). According to this proposal, in the social domain, the posterior cerebellum receives incoming information about others’ actions, checks the sequences of these actions against internal models, and sends back feedback to the cerebrum to signal whether the social action sequence is as predicted or not (Van Overwalle, Manto, et al., 2020b; Van Overwalle, Manto, et al., 2019b). This allows one to capture the temporal dynamics of social information and use it for future interactions, such as for smoothly anticipating others actions. For example, if you accidentally bump into someone and they fall, you might be worried that they are hurt (mentalizing) and therefore may check that they are okay, apologize, and help them up (interaction sequence).

Recent empirical evidence has supported this cerebellar function in social sequencing, demonstrating that the posterior cerebellar Crus 1 and 2 are involved in generating or memorizing sequences of social information, such as beliefs (Heleven & Van Overwalle, 2019; Van Overwalle, De Coninck, et al., 2019a), trait attribution (Pu et al., 2020, 2021), and social navigation (Li et al., 2021), both in an implicit or explicit manner (Ma, Pu, Haihambo, et al., 2021a; Ma, Pu, Heleven, et al., 2021b). The social sequence detection function of the posterior cerebellum also is supported by connectivity studies, which have demonstrated functional closed-loop connectivity (i.e., initiating and terminating in the same areas) from social mentalizing areas: for example, from the TPJ to the posterior cerebellar Crus 1 and 2, and back to the TPJ, and other mentalizing areas (Metoki et al., 2021; Van Overwalle, Van de Steen, & Mariën, 2019c; Van Overwalle, Van de Steen, et al., 2020c). The posterior cerebellar Crus 2 seems particularly sensitive to social actions that require mentalizing about beliefs, intentions, traits, and emotions of self and others compared with information that does not require mentalizing (see meta-analysis by Van Overwalle, Ma, & Heleven, 2020a). This is even more so when this information requires identifying the sequences of social actions as demonstrated in recent research on the social cerebellum mentioned above (Heleven & Van Overwalle, 2019; Li et al., 2021; Ma, Pu, Haihambo, et al., 2021a; Ma, Pu, Heleven, et al., 2021b; Pu et al., 2020, 2021; Van Overwalle, De Coninck, et al., 2019a).

Interestingly, earlier research on the social role of the cerebellum involved a variety of mentalizing inferences. The meta-analysis by Van Overwalle, Ma, and Heleven (2020a) mentioned earlier, covered a vast range of social information, going from low-level presence or intentions of social agents in concrete situations to high-level abstract inferences, such as traits. Likewise, an earlier meta-analysis on the involvement of the cerebellum in social cognition showed activation of the posterior cerebellum (mainly Crus 1 and 2) for different mentalizing inferences, ranging from low-level intentions and beliefs about concrete events, up to abstract traits summarizing qualities of persons and imaginary inferences about past/future events (which typically carry sequential information; Van Overwalle et al., 2014). In another study that pitted abstract trait inferences against concrete observations of the same behaviors, stronger activation of the posterior, cerebellar Crus 1 and 2 and mPFC was observed (Baetens et al., 2013).

### Present study

To our knowledge, almost all studies on the role of the cerebellum in processing sequential components of mentalizing involve the mere *detection* of sequences in social actions but did not investigate whether this information is used in *anticipating* upcoming social actions. One exception is the study by Haihambo et al. (2021) on the neural correlates involved in predicting future social action sequences based on personality traits (Haihambo et al., 2021). This study found that both cortical (mPFC, TPJ, and PCC) and cerebellar (Crus 1, 2, and lobule IX) mentalizing regions were involved when predicting sequences of events based on personality traits.

In the present study, we ask the question: Are the same mentalizing regions documented in prior cerebellar research on social action prediction based on high-level traits (Haihambo et al., 2021) also involved in predicting social action sequences based on less abstract, more temporary goal-directed social information such as intentions? In the previous study, social actions were described to the participants as being based on “personality traits” and included a wide variety of actions, such as (un)helpful, (un)kind, (dis)honest, (un)friendly, (un)just, (un)forgiving, (un)fair, anxious/calm, and so on. However, in the present study, actions were described as being based on “intentions” of (dis)honesty (using various synonyms). Because intentions to be honest (or not) generate temporary beliefs that are true (or false), they rely more on details about here-and-now information, rather than on abstract personality traits. For example, if someone’s personality is described as dishonest, this will define the characteristic of that person (i.e., that this person is generally dishonest). However, if someone has a dishonest intention, this will describe the goal of that person in a given situation.

Some studies have investigated mentalizing about intentions. For example, Atique et al. (2011) presented participants with cartoons depicting protagonist who had to choose one of two possible actions. For example, a child who is too small to reach a door handle, first reaches for a door handle and then for an umbrella. This sequence of actions assumes an intention to use the umbrella to open the door, not to use it in the rain. Although activation was found in mentalizing areas of the cerebrum (e.g., TPJ, mPFC, and precuneus), no activation was observed in the cerebellum. Similarly, Ciaramidaro et al. (2007) presented participants with cartoon images depicting a series of events leading up to a future social interactive intention (e.g., to plan a romantic date for someone who is not currently there) and participants had to choose which one of two options depicted the next logical step. They found activation in cerebral mentalizing areas (e.g., TPJ and precuneus) but did not include the cerebellum in their analysis. Presumably, activation in cerebellum in these studies was not observed, because the task did not specifically focus on the sequential nature of the intentions (i.e., no sequencing control condition) but rather on a choice between two goals. Of more interest for the present study, Grèzes et al. (2004) studied honest and deceitful intentions. They presented participants with videos of agents lifting a heavy or light box and participants had to judge if the actor was trying to deceive them about the weight of the box or not. The results showed that when participants judged intentions to be deceptive, there was activation in the TPJ and the cerebellar Crus 1 (although not in the mentalizing network demarcated by Buckner et al., 2011). Although informative, none of these studies investigated predictions of future actions. This still leaves open the question whether similar neural processes are involved when we think of a possible future logical sequence of events based on intention information.

**Fig. 1:**
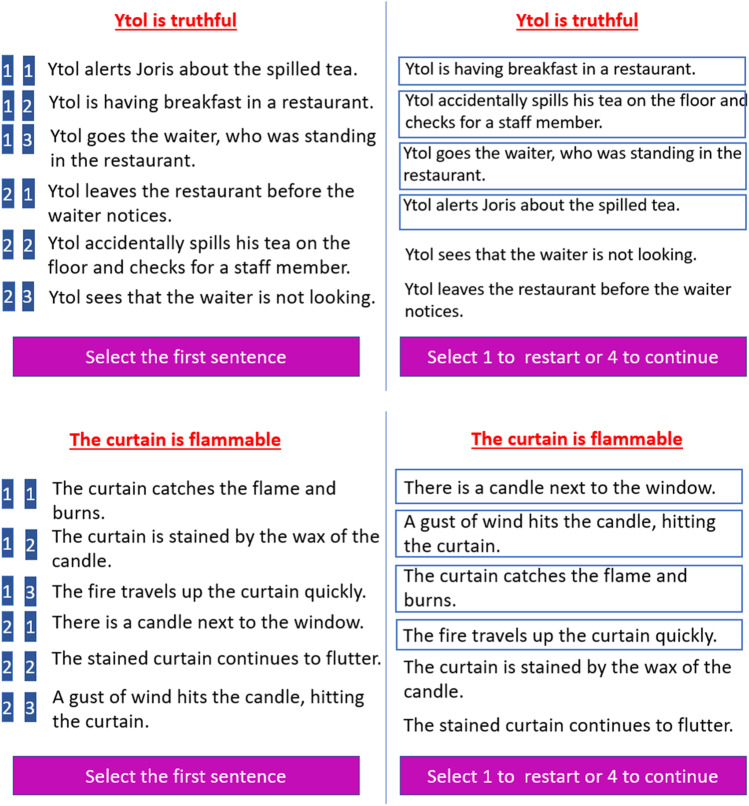
Illustration of a trial from the Social Sequencing (top panel) and Non-Social Sequencing (bottom panel) conditions. Left: Participants were presented six action sentences (randomly ordered) and were required to select the four sentences that fit best with the person intention / object feature, and to order them in the correct order (ignoring the inconsistent sentences) using two consecutive button presses on a four-button response box (with responses indicated on a blue background on the left of the screen). Right: The ordering as chosen by a participant (the four sentences were ordered from top to bottom in the order of selection and marked by squares surrounding them)

To investigate action prediction based on intentions, we adapted the prediction paradigm used in our previous prediction study involving personality traits (Haihambo et al., 2021) to present behavioral sentences that explicitly mentioned a protagonist’s intentions as being honest or deceitful (e.g., Ytol is honest), followed by a set of behavioral sentences related to these intentions (Fig. 1). We instructed our participants to predict the most plausible sequence of future actions that would follow given the protagonist’s intention. To verify that sequencing and social mentalizing were important components of cerebellar activation, we built in several control conditions: (a) a non-sequencing (Social Selection-only) task where participants merely selected the appropriate sentences based on intentions without giving a correct order, (b) a Non-social Sequencing task where social protagonists were replaced with inanimate objects, and (c) a combined Non-social Selection-only task.

Similar to our previous findings on traits (Haihambo et al., 2021) and in line with the cerebellar sequencing hypothesis (Leggio & Molinari, 2015), our primary hypothesis is that cerebellar mentalizing areas are activated in social action prediction based on intentions (compared to the Selection-only and the Non-social control conditions). However, given the lower abstraction level of intentions, it is possible that not all areas recruited in the prior high-level trait prediction study will be activated (i.e., posterior Crus 1 and 2, and anterior lobule IX) or that additional cerebellar areas will be activated. We also hypothesized parallel activations in the mentalizing areas of the cerebrum (TPJ, mPFC, and precuneus/PCC) in line with the earlier prediction study showing these activations (Haihambo et al., 2021). Because the cerebral cortex is less sensitive to sequencing (Caligiore et al., 2019); however, we expected these activations regardless of sequencing, that is, during sequencing as well as during social selection-only manipulations (i.e., secondary mentalizing hypothesis).

## Method

### Participants

Participants in this study were 26 healthy, right-handed, Dutch-speaking volunteers (10 males: mean age 23 years old, standard deviation [SD] = 3 years). This number is similar to the previous study (n = 27; Haihambo et al., 2021). Participants all had normal or corrected-to-normal vision and reported no neurological or psychiatric disorders. Informed consent was obtained following the guidelines of the Medical Ethics Committee at the Gent University Hospital, where the study was conducted. Participants were given 20 euros and reimbursed for transportation costs in exchange for their participation.

### Stimulus material

The present study used the social action prediction paradigm adjusted from the previous study by Haihambo et al. (2021). In the present task, participants were presented with social and non-social sentence sets. The social sentence sets consisted of a prompt sentence and six intention implying sentences. Unlike Haihambo et al. (2021) who manipulated a whole range of traits related to ability and sociality, the intentions of the main protagonist in this study were restricted to honesty and deceit (Grèzes et al., 2004) in order to create true and false beliefs in the responding protagonist. In particular, in the first prompt sentence, participants were presented with a protagonist and a short description of their truthful or misleading intention (e.g., Ytol is honest, Mata is cunning, etc.). The names used in these prompt sentences were fictional Star-Trek-like names and synonyms of deceptive or honest were used as intentions to provide variety. This was followed by six behavioral sentences that described interactions between two or more protagonists. These six sentences were made up of, two neutral, two consistent, and two inconsistent sentences relative to the intention presented in the prompt sentences (Fig. 1A). The two inconsistent sentences served as distractors, because they were not part of the correct answer.

The non-social control sentence sets consisted of a prompt sentence involving an object and followed by six sentences that implied the same characteristic of the object. Specifically, in the first prompt sentence, participants were presented with an object and its characteristics (e.g., the curtain is flammable). This prompt sentence was followed by six non-social sentences that were made up of two neutral, two consistent, and two inconsistent sentences relative to the object characteristic presented in the prompt sentence (Fig. 1B).

These social and non-social sentences were randomly distributed between two tasks: a Sequencing task and a Selection-only task (non-sequencing control). This resulted in a Domain (Social vs. Non-social) by Task (Sequencing vs. Selection-only) design consisting of four conditions: Social Sequencing, Social Selection-only, Non-social Sequencing, and Non-social Selection-only. The sentence structures were identical between conditions, with the exception that the social conditions included social agents performing social actions, whereas the Non-social conditions included non-social objects in relation to their environment. All sentences were either newly developed for this study or adapted from the verbal false belief sequencing task by Heleven et al. (2019). All social and non-social sentences were pilot tested for sequencing accuracy. In the pilot study, participants (n = 47) were presented with the six action sentences and were instructed to put them in the correct chronological order and also were asked how consistent a sentence was to the intention on a 7-rating scale with anchors: 1 = inconsistent, 4 = neutral, 7 = consistent. Sentence sets were included if they were sequenced correctly at least 65% of the time and received a mean consistency score of 3.5-4.5 for neutral sentences, <3.5 for inconsistent sentences, and >5.5 for consistent sentences. The sequencing accuracy score of minimally 65% allowed for great variation in difficulty. Participants in this pilot study did not participate in the fMRI study.

### Procedure

The procedure of this study is identical to the one used in the previous trait predicting task (Haihambo et al., 2021). Participants were instructed that the experiment included two tasks: Sequencing and Selection-only, which they would perform in this order. To avoid spill-over learning effects, the Selection-only task always came last, whereas the social and non-social sentences were randomized within each task. In the sequencing task, participants were instructed to “select only the four sentences that fit the intention of the person or characteristic of the object and put them in the correct chronological order.” In the Selection-only task, they were told that “the sentences are now already put in the correct order” and that they only had to “select the four sentences that fit the intention of the person or characteristic of the object” without generating the correct order. They were further told to “execute this task correctly and as quickly as possible. Your time is measured from the presentation of the event until you indicate that you are ready.”

In each trial of the Sequencing task (Fig. 1A), participants were first shown an intention of a protagonist (social condition) or a characteristic of an object (Non-social condition). This prompt sentence appeared in red on the top of the screen, remaining there for the entire duration of the trial. After 1,000 ms, the first of six sentences were shown on the screen followed by the remaining five sentences, which appeared one-by-one after 1,300 ms each, which is based on previous trait prediction study (Haihambo et al., 2021), because the procedure and sentence length were comparable. The six sentences were presented in random order for each participant and for each trial. After individual presentation, all sentences were shown together on screen in the same random order along with numbers on the side of the sentences (Fig. 1). Then, a prompt to select the first sentence appeared at the bottom of the screen, followed by a prompt to select the next sentence, until they selected all sentences. No duration was set for completing this task. Once four sentences were selected, participants were then prompted to select “1 to restart or 4 to continue.” At the end of each trial, a confidence question appeared: “how confident are you about your answer” and a 4-point rating scale ranging from 1 = not at all to 4 = very much. Participants responded with a button press using an MRI safe button response box. All trials and confidence ratings were preceded by a blank screen with a fixation cross, jittered randomly between 1–2 s. The same procedure was used for both Social and Non-social conditions.

In the Selection-only task, the procedure was identical to the sequencing task, with the exception that participants did not have to put the sentences into the correct chronological order and individual sentences were presented one-by-one for 1,100 ms (as in the previous trait prediction study) instead of 1,300 ms used for sequencing. During piloting, participants reported that they needed more time in the Sequencing task than in the Selection-only task, resulting in 200 ms being added to the Sequencing task. While this 200-ms difference represents a potential confound in presentation duration, it avoids a confound at a psychological level (i.e., undue waiting during Selection-only or rushed reading during the presentation sentences during the sequencing tasks). Additionally, participants were presented with two neutral sentences in their correct chronological order, followed by a pair of consistent or inconsistent sentences each in their correct chronological order, and not randomly as in the Sequencing task. Participants had to select only one of these two sentence sets by selecting “1” or “2,” followed by a confidence rating as in the sequencing task. The entire experiment lasted approximately 45 minutes.

Before entering the scanner, participants were presented with a short practice version of the experiment. They were presented with three sequencing and three selection-only trials that were not part of the fMRI experiment, followed by confidence ratings. This allowed participants to practice the response presses and order the sentences. The whole experiment outside and inside the scanner was presented in E-Prime 3.0 (www.pstnet.com/eprime; Psychology Software Tools), running on a Windows 10 and Windows XP computer respectively. In the scanner, participants used a four button MR compatible response box positioned in their left hand.

In total, participants completed 44 trials, each consisting of 6 different sentences. Each Sequencing or Selection-only task consisted of 11 social and 11 non-social trials. Participants first received the Sequencing task, after which they received the Selection-only task. This was done so that participants were not primed with the correct structure of already ordered sentences in the Selection-only task. For each task, the social and non-social trials (i.e., sentence sets) were presented in a random order for each participant.

### Imaging procedure and pre-processing

Images were collected with a Siemens Magnetom Prisma fit scanner system (Siemens Medical Systems, Erlangen, Germany) using a 64-channel radiofrequency head coil. Stimuli were projected onto a screen at the end of the magnet bore, which participants viewed by way of a mirror mounted on the head coil. Participants were placed headfirst and supine in the scanner bore and were instructed not to move their heads to avoid motion artifacts. Foam cushions were placed within the head coil to minimize head movements. First, high-resolution anatomical images were acquired using a T1-weighted 3D MPRAGE sequence [repetition time (TR) = 2,250 ms, echo time (TE) = 4.18 ms, inversion time (TI) = 900 ms, field of view (FOV) = 256 mm, flip angle = 9°, voxel size = 1 × 1 × 1 mm]. Second, a fieldmap was calculated to correct for inhomogeneities in the magnetic field (Cusack & Papadakis, 2002). Third, whole brain functional images were collected in a single run using a T2*-weighted gradient echo sequence, sensitive to blood oxygen level dependent (BOLD) contrast (TR = 1,000 ms, TE = 31.0 ms, FOV = 210 mm, flip angle = 52°, slice thickness = 2.5 mm, distance factor = 0%, voxel size = 2.5 × 2.5 × 2.5 mm, 56 axial slices, acceleration factor GeneRalized Autocalibrating Partial Parallel Acquisition (GRAPPA) = 4).

SPM12 (Wellcome Department of Cognitive Neurology, London, UK) was used to process and analyze the fMRI data. To remove sources of noise and artifacts, data were preprocessed. Functional data was corrected for differences in acquisition time between slices for each whole-brain volume, realigned to correct for head movement, and co-registered with each participant’s anatomical data. Then, the functional data was transformed into a standard anatomical space (2-mm isotropic voxels) based on the ICBM152 brain template (Montreal Neurological Institute). Normalized data were then spatially smoothed (6-mm full width at half-maximum, FWHM) using a Gaussian Kernel. Finally, using the Artifact Detection Tool (ART; http://web.mit.edu/swg/art/art.pdf;http://www.nitrc.org/projects/artifact_detect), the data were examined for excessive motion artifacts and for correlations between motion and experimental design, and between global mean signal and experimental design. Outliers were identified in the temporal differences series by assessing between-scan differences (Z-threshold: 3.0 mm, scan to scan movement threshold: 0.5 mm; rotation threshold: 0.02 radians). These outliers were omitted from the analysis by including a single regressor for each outlier. A default, high-pass filter was used of 128 s and serial correlations were accounted for by the default auto-regressive (AR) model.

### Statistical analysis of behavioral data

Accuracy for both sequencing and Selection-only tasks were calculated by giving 1 point for a correct selection and 0 points for an incorrect response. So, the maximum score on a Sequencing trial is 4 and 1 for a Selection-only trial. The response time (RT) was calculated by timing the whole trial: i.e., starting after all six sentences were all presented on screen for the first time and the prompt to select or sequence the sentences appeared, until they selected the final sentence.

A repeated measure analysis of variance (ANOVA) with Domain (Social vs. Non-social) and Task (Sequencing vs. Selection-only) as within-participants factors was conducted on accuracy and RT using ISM SPSS Statistics 27 software. The alpha level for pairwise comparisons was set at 0.05, corrected for multiple comparisons using Bonferroni correction and is reported when significant interactions were revealed.

### Statistical analysis of neuroimaging data

At the first (single participant) level, for each task, the event-related design was modelled with a separate regressor for each condition (i.e., Social Sequencing, Non-social Sequencing control, Social Selection-only control, and Non-social Selection-only control). The onset of each trial was set after all six sentences were presented together on screen and the prompt to select or sequence the sentences appeared. The presentation of each sentence was relatively short, so that little time was left for anything else other than reading. Hence, although participants could start eliminating inconsistent sentences as soon as they saw one sentence, properly sequencing the sentences was only possible after all sentences were carefully read. Based on considerations of how response processes might have evolved during a trial and our goal to select equivalent timings for fMRI analysis across conditions, duration was set from the onset of the trial (i.e., when the prompt sentence appeared) until the time participants made their final selection (i.e., selection of four sentences reflecting the assumed intent in the Sequencing and the Selection-only tasks). This timing reflects the same process across the two tasks. All trials were analyzed, irrespective of whether selection or sequencing was correct, because we assumed that participants’ selection and sequencing was based on what they believed to be correct. When a trial was canceled and redone, analysis was performed on the responses and timing of the final sentence selection.

At the second (group) level, a whole-brain random effects analysis using one-way within-participants ANOVA was used. Significance was set at the cluster-defining uncorrected threshold of *p* < 0.001, followed by a cluster-wise FWE corrected threshold *p* < 0.05, with a minimum cluster extent of 10 voxels. We also tested our hypotheses more directly by performing a Region of Interest (ROI) analysis, using spheres centered on *a priori* MNI coordinates for the cerebellar Crus 1 and 2 (±40 −70 −40 and ±24 −76 −40 respectively; Van Overwalle, Ma, & Heleven, 2020a) and a 15-mm radius. ROI analyses were done using a small volume (rather than whole-brain volume) correction for multiple comparisons with the same thresholds as whole-brain analysis. To avoid redundancy, however, we report ROI results only when whole-brain contrasts were not significant (i.e., denoted by “ROI” in the tables).

## Results

### Behavioral results

A repeated ANOVA with Domain (Social and Non-social) and Task (Sequencing and Selection-only) within-participants factors was conducted on accuracy and RT.

#### Accuracy

Results of the repeated measures ANOVA revealed significant main effects of Domain (*F* (1, 26) = 8.34, *MSE =* 0.005*, p* = 0.008, ηp^2^ = 0.24) and Task (*F* (1, 26) = 8.53, *MSE* = 0.007*, p* = 0.008, ηp^2^ = 0.25), indicating that participants were more accurate in the social conditions (Sequencing: *M* = 82%, *SD* = 24%; Selection-only: *M* = 93%, *SD* = 8%) than in the Non-social conditions (Sequencing: *M* = 77%, *SD* = 24%; Selection-only: *M* = 90%, *SD* = 13%), but their interaction was not significant (*p* = 0.56).

#### RT

The results revealed significant main effects for Domain (*F* (1, 26) = 34.26, *MSE* = 18.58*, p* < 0.001, ηp^2^ = 0.60) and Task (*F* (1, 26) = 178.89, *MSE* = 181.74*, p* < 0.001, η_p_^2^ = 0.87), indicating that participants were faster in the non-social conditions (Sequencing: *M* = 49 s, *SD* = 17 s; Selection-only: *M* = 15 s, *SD* = 9 s) than in the Non-social conditions (Sequencing: *M* = 45 s, *SD* = 16 s; Selection-only: 10 s, *SD* = 7 s) but no significant interaction (*p* = 0.33).

Generally, our results indicate that participants performed faster on social than non-social material.

 Note that although we included confidence ratings during our task, scores were generally high (i.e., 4/4), and so no further behavioral or fMRI analysis was performed including these scores.

### fMRI results

To investigate the social and sequencing functions of the cerebellum, we computed a number of contrasts comparing Social versus Non-social conditions and Sequencing versus Selection-only conditions, while holding the other manipulations constant. Recall that we hypothesized stronger activation in cerebellar and cortical mentalizing areas given social as opposed to non-social conditions, and additionally stronger cerebellar activation in sequencing as opposed to Selection-only conditions. Additionally, we report the reverse contrasts to exhaustively test that the hypothesized effects are found only in the expected direction of the comparison. For ease of presentation, we describe all activations in the cerebellum and mentalizing areas of interest and only peak activations in other areas.

#### Social sequencing versus social selection-only

**Fig. 2:**
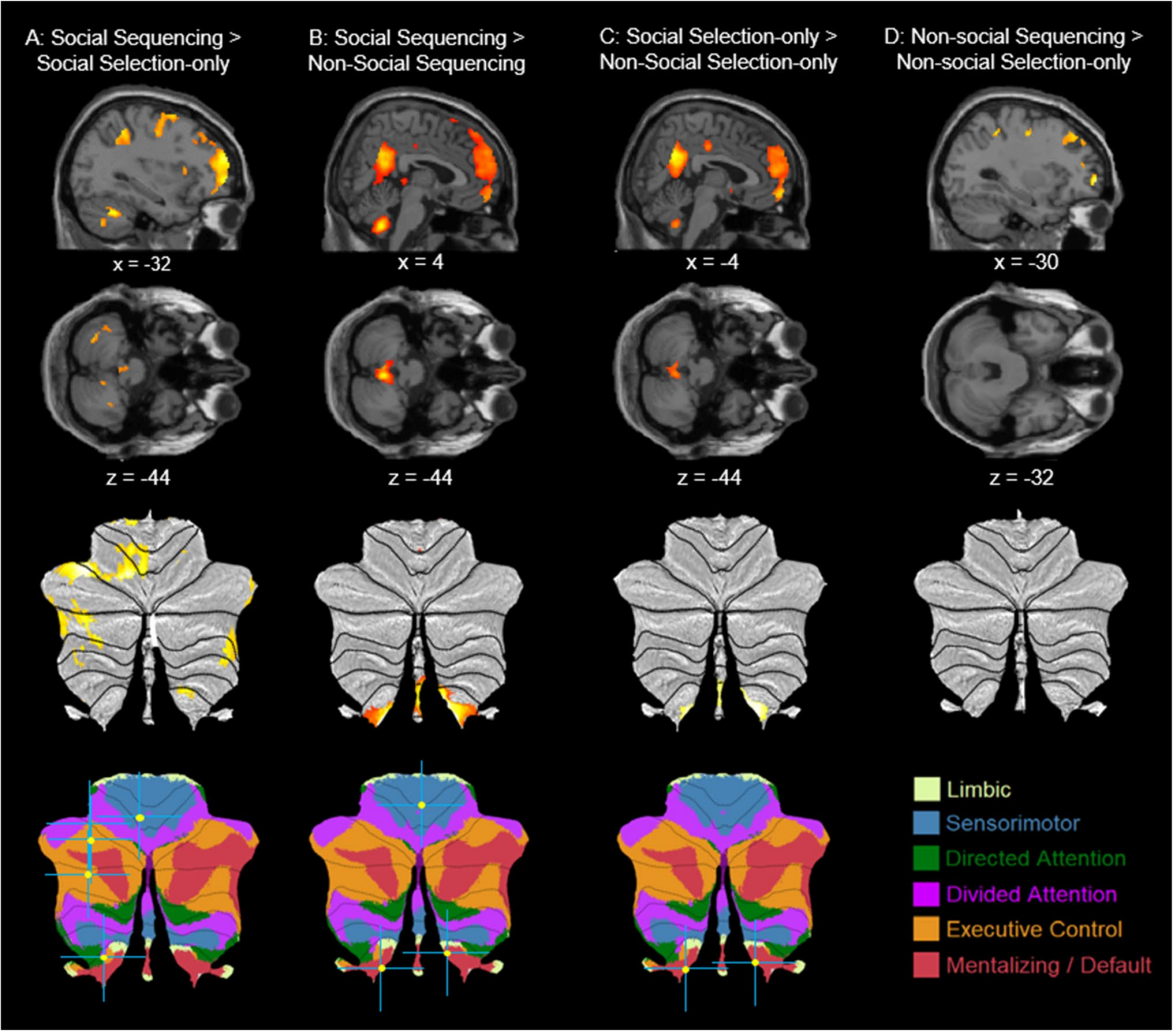
Sagittal and transverse views of the experimental contrasts involving sequencing and Selection-only conditions, visualized at a whole-brain uncorrected threshold of ***p*** < 0.001, together with visualization on SUIT flatmaps of the cerebellum. A Social Sequencing > Social Selection-only contrast showing Crus 1 and lobule IX activation, **B** Social Sequencing > Non-social Sequencing contrast showing lobule IX activation, and **C** Social Selection-only > Non-social Selection-only contrast, showing lobule IX activation, and **D** Non-social Sequencing > Non-social Selection-only contrast**,** showing no cerebellar activation. Peak activations of significant contrasts also are indicated with a yellow circle and a blue crosshair on the 7-network structure from Buckner, Krienen, Castellanos, Diaz, and Yeo (2011) shown on a cerebellar flatmap (http://www.diedrichsenlab.org/imaging/AtlasViewer/viewer.html)

Consistent with our primary hypothesis, a Social Sequencing > Social Selection-only contrast (Fig. 2A; Table 1) revealed activations in the left cerebellar lobule IV and IX. Further activations were observed in the left lingual gyrus, left inferior parietal lobe, left intraparietal sulcus (IPS), right mid-cingulate cortex (MCC), bilateral superior frontal gyrus, superior medial gyrus, caudate nucleus, and anterior cingulate cortex (ACC). A ROI analysis of the contrast revealed activations in the left cerebellar Crus 1. The opposite contrast (Social Sequencing < Social Selection-only) only revealed activation in the left insula.

**Table 1:** Whole-brain analysis contrasting Social Sequencing against Social Selection-only and social versus Non-social Sequencing

Brain label/contrast	MNI coordinates			Voxels	Max t
	x	y	z		
Social sequencing > Social Selection-only					
ROI: L cerebellum Crus 1	-32	-64	-44	38	4.49**
L cerebellum VII	-34	-58	-36		3.78*
L cerebellum VI	-34	-56	-32	169	5.57*
L cerebellum VII	-32	-64	-44		4.49
L cerebellum VI	-26	-64	-26		3.69
L lingual gyrus	-18	-70	-10	424	6.25**
L cerebellum IX	-20	-42	-36		4.66
L cerebellum VI	-6	-62	-14		4.25
L inferior parietal lobule	-32	-44	48	333	4.96
Area hIP1 IPS	36	-42	42	268	5.95**
R cingulate gyrus	6	-30	28	611	6.75***
R superior frontal gyrus	24	2	56	4065	7.86***
L superior frontal gyrus	-20	10	68	1286	6.95***
L superior medial gyrus	-6	24	42	318	5.31*
L caudate nucleus	-16	24	-8	430	5.14
R ACC	8	30	28	127	4.47
L middle frontal gyrus	-34	56	20	1878	6.32**
Social Sequencing < Social Selection-only					
L insula lobe	-30	-16	10	108	4.58
Social Sequencing > Non-social Sequencing					
R cerebellum IX	4	-56	-44	530	9.07***
L cerebellum IX	-6	-54	-42		7.14***
L PCC	-4	-48	30	1789	11.34***
Cerebellar vermis 4/5	2	-56	4		3.68
L angular gyrus, including TPJ	-42	-52	24	181	7.87***
R thalamus	8	-30	4		5.14*
L hippocampus	-24	-24	-12	881	5.92**
R hippocampus	22	-8	-16	559	6.16**
L medial temporal pole	-52	8	-28	3057	10.59***
R middle temporal gyrus	58	-6	-16	2232	13.39***
L middle frontal gyrus	-26	18	40	417	5.25*
R IFG p. triangularis	52	22	18	114	4.42
L rectal gyrus, including mPFC	0	52	-16	4909	9.11***
L superior medial gyrus	-8	60	30		8.99***
L superior medial gyrus	-10	32	58		8.13***
Social Sequencing < Non-social Sequencing					
L precentral gyrus	-46	4	18	109	4.89
L IFG p. triangularis	-40	34	20	423	6.07**
R middle frontal gyrus	48	46	14	291	5.3*

Coordinates refer to the MNI (Montreal Neurological Institute) stereotaxic space reported for all (sub)clusters in cerebellar and mentalizing areas, and only for peak clusters for other areas. Whole-brain analysis thresholded at cluster-defining uncorrected *p* < 0.001 and cluster-wise FWE corrected *p* < 0.05 and voxel extent ≥10. L = left, R = right, PCC = posterior cingulate cortex, TPJ = temporoparietal junction, mPFC = medial prefrontal cortex

*p < 0.05; **p < 0.01; ***p < 0.001 (peak FWE corrected)

#### Social Sequencing versus Non-social Sequencing

A Social Sequencing > Non-social Sequencing contrast (Fig. 2B, Table 1) revealed, consistent with our hypotheses, robust activations in the bilateral Lobule IX in the cerebellum, along with cortical activations in other mentalizing areas, including the left PCC, left angular gyrus (including TPJ), left rectal gyrus, including the mPFC and the left orbital frontal cortex (OFC). We also found activations in the bilateral hippocampus, bilateral medial temporal pole, left middle frontal gyrus, and right inferior frontal gyrus (IFG) triangularis. However, contrary to our primary cerebellar hypothesis, neither whole brain nor ROI analysis revealed activation of cerebellar Crus 1 or 2 in this (or the opposite) contrast. The opposite contrast (Non-social Sequencing > Social Sequencing) revealed activations in the left IFG pars triangularis, right middle frontal gyrus and left precentral gyrus.

#### Social Selection-only versus Non-social selection-only

A Social Selection-only > Non-social Selection-only contrast (Fig. 2C; Table 2) revealed activations in the bilateral Lobule IX in the cerebellum and, consistent with our secondary mentalizing hypothesis, cortical activations in mentalizing areas, including the angular gyrus (including TPJ), bilateral PCC, including precuneus, and left rectal gyrus, including the mPFC and OFC. We found further activations in the bilateral hippocampus, MCC, right temporal pole and left middle frontal gyrus. Neither whole brain nor ROI analysis reveal activation of cerebellar Crus 1 or 2 in this (or the opposite) contrast. The opposite contrast (Non-social Selection-only > Social Selection-only) revealed activations in the left temporal pole, inferior partial lobe, bilateral IFG pars opercularis, left IFG triangularis, and right middle frontal gyrus.

**Table 2:** Whole-brain analysis comparing Social versus Non-social Selection-only and Non-social Sequencing versus Non-social Selection-only

Brain label/contrast	MNI coordinates			Voxels	Max t
	x	y	y		
Social Selection-only > Non-social Selection-only					
R cerebellum IX	4	-54	-44	151	5.01
L cerebellum IX	−4	-54	-42		4.68
L angular gyrus	-44	-52	22	137	6.52***
L PCC	−2	-50	30	1476	9.05***
R PCC	12	-44	30		5.25*
R precuneus	6	-52	12		4.73
L hippocampus	-24	-24	-12	354	5.32*
R MCC	4	-20	40	179	4.82
L middle temporal gyrus	-58	-12	-10	2131	8.88***
R hippocampus	20	-8	-18	243	5.07
R temporal pole	44	18	-30	1854	10.89***
L middle frontal gyrus	-24	22	44	112	4.37
L rectal gyrus, including mPFC	−2	50	-18	2514	7.9***
Social Selection-only < Non-social Selection-only					
L Middle Temporal Gyrus	-48	-56	−2	356	5.44*
L inferior parietal lobule	-38	-48	38	121	4.67
L IFG p. opercularis	-46	8	24	340	5.48*
R IFG p. opercularis	48	10	24	150	4.89
L IFG p. triangularis	-44	34	18	1552	9.04***
R middle frontal gyrus	44	34	20	1129	5.89**
Non-social Sequencing > Non-social Selection-only					
R cingulate gyrus	2	-30	28	1064	5.14
L precentral gyrus	-36	−8	48	614	4.86
R superior frontal gyrus	24	2	58	1709	6.76***
L caudate nucleus	-10	6	-10	170	4.81
L middle frontal gyrus	-22	30	40	284	4.55
Non-social Sequencing < Non-social Selection-only					
L inferior temporal gyrus	-38	-54	0	108	4.49
Subiculum	24	8	24	168	5.4*
L IFG p. orbitalis	-36	36	-8	190	4.78

Coordinates refer to the MNI (Montreal Neurological Institute) stereotaxic space reported for all (sub)clusters in cerebellar and mentalizing areas, and only for peak clusters for other areas. Whole-brain analysis thresholded at cluster-defining uncorrected p < 0.001 and cluster-wise FWE corrected *p* < 0.05, with voxel extent ≥10. L = left, R = right, TPJ = temporoparietal junction, mPFC = medial prefrontal cortex, PCC = posterior cingulate cortex, MCC = mid-cingulate cortex, IFG = inferior frontal gyrus

*p < 0.05; **p < 0.01; ***p < 0.001 (peak FWE corrected)

#### Non-social Sequencing versus Non-social Selection-only

To validate the specificity of cerebellar involvement in Social Sequencing (as opposed to non-social scenarios), we also computed a Non-social sequencing > Non-Social Selection-only contrast (Fig. 2D; Table 2). We found activation in the cingulate gyrus, left precentral gyrus, right superior frontal gyrus, left caudate nucleus, and left middle frontal gyrus. As one would expect given a lack of social or sequencing manipulation, neither whole-brain nor ROI analysis revealed any cerebellar activation. The opposite contrast revealed activation in the subiculum and left IFG pars orbitalis.

#### Interaction

**Fig. 3:**
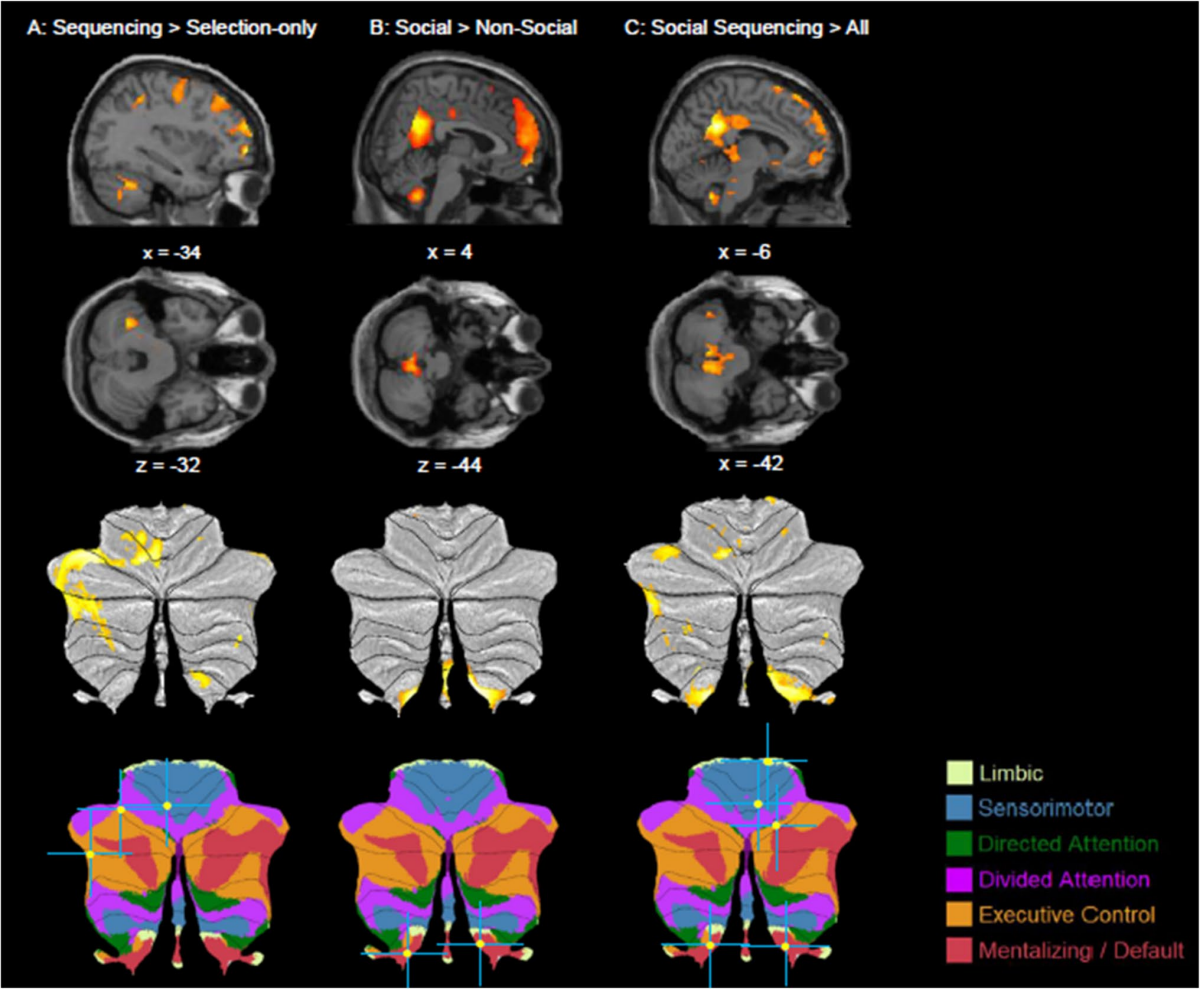
Sagittal and transverse views of interaction results of sequencing and social contrasts, visualized at a whole-brain uncorrected threshold of ***p*** < 0.001, together with visualization on SUIT flatmaps of the cerebellum.** A** Sequencing (Social Sequencing > Non-social Sequencing) > Selection-only (Social Selection-only > Non-social Selection-only) showing cerebellar activation, **B** Social (Social Sequencing > Social Selection-only) > Non-social (Non-social Sequencing > Non-social Selection-only) contrast showing lobule IX activation. **C** Asymmetric interaction analysis of Social Sequencing > All (Social Selection-only, Non-social Sequencing, Non-social Selection-only) showing cerebellar activation. Peak activations of significant contrasts are also indicated with a yellow circle and a blue crosshair on the 7-network structure from Buckner, Krienen, Castellanos, Diaz, and Yeo (2011) shown on a cerebellar flatmap (http://www.diedrichsenlab.org/imaging/AtlasViewer/viewer.html)

To confirm our hypothesis that the posterior cerebellum is involved in Social Sequencing specifically, we computed an asymmetric interaction contrasting Social Sequencing against all other conditions. Specifically, we set a positive contrast weight of +3 for the Social Sequencing condition and a negative contrast weight of −1 for all three other conditions (Social Selection-only, Non-social Selection-only, and Non-social Sequencing; Fig. 3C; Table 3). This asymmetric interaction is a more adequate test of our hypothesis than a typical crossover interaction in which both Social Sequencing and Non-social Selection-only conditions would receive positive weights, and the other conditions negative weights. Results show activations in cerebellar mentalizing areas in lobule IX, along with limbic areas and sensorimotor areas in the anterior lobules IV-V and VI. We found additional cortical activations in the PCC, bilateral parahippocampal gyrus, bilateral temporal pole, left caudate gyrus and bilateral superior frontal gyrus. The opposite contrast (with a weight of +3 for non-social sequencing and −1 for all other conditions) revealed activations in the MCC, bilateral superior frontal gyrus and left middle frontal gyrus. To be exhaustive, we also conducted a crossover analysis ((Social Sequencing > Social Selection-only) > (Non-social Sequencing > Non-social Selection-only)); however, as one would expect, neither whole-brain nor ROI analysis revealed any activations.

**Table 3:** Whole-brain analysis showing asymmetric interactions analysis for Social and Non-social Sequencing

Brain label/contrast	MNI coordinates			Voxels	Max t
	x	y	y		
Asymmetric interaction: Social Sequencing > all other conditions (Social Selection-only, Non-social Sequencing, Non-social Selection-only)					
L cerebellum IX	−6	−54	−42	419	5.40*
R cerebellum IX	6	-52	−46		5.09*
L PCC	−-4	−48	28	3212	8.47***
R parahippocampal gyrus	26	-18	-22		6.44***
R MCC	12	-48	32		6.18**
L parahippocampal gyrus	8	-22	-22	222	5.38*
L cerebellum IV-V	10	-32	-26		5.11*
L cerebellum VI	-20	-70	-14	122	5.21*
L parahippocampal gyrus	-12	-24	-24	114	5.94**
R temporal pole	42	18	-30	1128	8.29***
R middle temporal gyrus	58	-6	-16		8.04***
R middle temporal gyrus	56	4	-26		7.00***
L claudate nucleus	-16	24	-8	412	5.58*
L temporal pole	-38	20	-32	1017	6.63***
L middle temporal gyrus	-56	0	-20		6.60***
L middle temporal gyrus	-52	6	-28		6.41***
L superior frontal gyrus	-28	62	20	6075	6.21**
R superior frontal gyrus	24	2	56		6.11**
R superior frontal gyrus	24	14	64		5.69**
Asymmetric interaction: Non-social Sequencing > all other conditions (Social Selection-only, Non-social Sequencing, Non-social Selection-only)					
R MCC	4	-30	26	415	5.56*
L MCC	-10	-28	38	105	5.61**
R superior frontal gyrus	26	4	60	829	6.81***
R superior frontal gyrus	20	6	66		5.46*
L superior frontal gyrus	-20	10	68	634	5.75**
L superior frontal gyrus	-20	2	62		5.65**
L paracentral gyrus	-24	-10	48		5.23*
L middle frontal gyrus	-28	58	2	68	5.34*

Coordinates refer to the MNI (Montreal Neurological Institute) stereotaxic space reported for all (sub)clusters in cerebellar and mentalizing areas, and only for peak clusters for other areas. For contrast weights in these interaction effects, see text. Whole-brain analysis thresholded at cluster-defining uncorrected *p* < 0.001 and cluster-wise FWE corrected *p* < 0.05, with voxel extent ≥10. L = left, R = right, mPFC = medial prefrontal cortex, PCC = posterior cingulate cortex, MCC = mid-cingulate cortex

*p < 0.05; **p < 0.01; ***p < 0.001 (peak FWE corrected)

#### Sequencing versus Selection-only main effect

To additionally investigate the Social Sequencing specificity of the cerebellar function, we compared all sequencing versus selection contrasts irrespective of (non)social conditions. First, we computed a main effect analysis in which we compared the sequencing conditions against the Selection-only conditions. Specifically, we set positive contrast weights to +1 for Social Sequencing and Non-social Sequencing conditions, and negative −1 weights for Social Selection-only and Non-social Selection-only conditions. In this Sequencing versus Selection-only main effect analysis (Fig. 3A; Table 4), we found activations in the left lingual gyrus, cerebellar lobule VI and Crus 2, left inferior parietal lobe, MCC, bilateral superior frontal gyrus, left rectal gyrus, right caudate, and bilateral middle frontal gyrus. Neither whole brain nor ROI analysis revealed activations in the opposite contrast.

**Table 4:** Whole-brain analysis showing sequencing and social main effects

Brain label/contrast	MNI coordinates			Voxels	Max t
	x	Y	y		
Main effect: Sequencing > Selection-only					
L lingual gyrus	−18	−70	−10	369	6.17**
L cerebellum VI	−6	−62	−14		4.95*
L cerebellum VI	−34	−56	−32	246	5.35**
L cerebellum crus 2	−44	−54	−44		4.57°
L inferior parietal lobule	−32	−44	48	137	5.61**
Area 2	28	−44	46	201	5.90**
L MCC	4	−30	26	1523	7.28***
L MCC	0	−22	28		5.69**
L MCC	−8	−28	38		5.48**
L superior frontal gyrus	−16	10	70	1302	6.23**
L superior frontal gyrus	−28	0	66		5.87**
L middle frontal gyrus	−24	−8	48		5.71**
R superior frontal gyrus	26	4	60	2732	8.64***
R superior frontal gyrus	24	14	62		7.62***
R middle frontal gyrus	30	42	44		6.51**
L rectal gyrus	−16	24	−8	495	5.61**
R caudate nucleus	16	24	−8	118	5.10*
L middle frontal gyrus	−42	26	44	628	5.02*
L middle frontal gyrus	−34	32	46		4.77*
L middle frontal gyrus	−50	24	34		4.75*
L middle frontal gyrus	−28	58	2	555	7.29**
L superior frontal gyrus	−28	62	20		5.82**
L middle frontal gyrus	−38	50	16		5.51**
Main effect: Sequencing < Selection-only					
---					
Main effect: Social > Non-social					
R cerebellum IX	4	−54	−44	364	7.36***
L cerebellum IX	−6	−54	−42		7.03***
L angular gyrus	−42	−52	24	188	8.31***
Angular gyrus	40	−48	22	117	6.35***
L PCC	−4	−48	30	1895	12.08***
L hippocampus	−24	−24	−14	418	6.19***
R hippocampus	26	−18	−18	395	6.10***
R MCC	2	−20	40	137	5.17***
R middle temporal gyrus	54	−8	−16	1985	13.15***
R medial temporal pole	44	16	−32		12.82***
R middle temporal gyrus	54	−24	−8		7.47***
L middle temporal gyrus	−56	2	−20	2567	10.48***
L medial temporal pole	−50	8	−28		10.44***
L middle temporal gyrus	−60	−12	−12		10.15***
R mid orbital gyrus, including mPFC	0	52	−12	3926	8.38***
L superior medial gyrus	−10	62	32		7.36***
R ACC	6	52	14		7.15***
Main effect: Social < Non-social					
L Middle Temporal Gyrus	−48	−56	-2	398	6.63***
L Inferior Parietal Lobule	−54	−32	38	331	6.22**
L IFG p. Opercularis	−44	6	24	295	5.94**
L Insula Lobe	−38	0	2	134	5.28*
L IFG p. triangularis	−44	34	18	691	7.70***
L IFG p. triangularis	−44	40	8		6.13**
L IFG p. triangularis	−50	34	24		5.81**

Coordinates refer to the MNI (Montreal Neurological Institute) stereotaxic space reported for all (sub)clusters in cerebellar and mentalizing areas, and only for peak clusters for other areas. For contrast weights in these main effects, see text. Whole-brain analysis thresholded at cluster-defining uncorrected *p* < 0.001 and cluster-wise FWE corrected *p* < 0.05, with voxel extent ≥10. L = left, R = right, mPFC = medial prefrontal cortex, PCC = posterior cingulate cortex, MCC = mid-cingulate cortex, IFG = inferior frontal gyrus

°*p* < 0.08; *p < 0.05; **p < 0.01; ***p < 0.001 (peak FWE corrected)

#### Social versus Non-social main effect

Additionally, we computed a similar main effect analysis contrasting the social versus non-social conditions. Specifically, we set positive contrast weights of +1 for Social Sequencing and Social Selection-only conditions and negative −1 weights for Non-social Sequencing and Non-social Selection-only conditions. In this social > Non-social main effect (Fig. 3B; Table 4), we found activations in the cerebellar lobule IX, left angular gyrus, posterior cingulate cortex, hippocampus, MCC, bilateral middle temporal gyrus, and the mid orbital gyrus including the mPFC. The ROI analysis revealed no further activations in posterior cerebellar areas. The opposite contract revealed activations in the left middle temporal gyrus, left inferior parietal lobe, IFG p. opercularis, left insula lobe, and IFG p. triangularis.

## Discussion

Mentalizing about the intentions of others is an important social skill, because it allows us to engage in smooth and efficient interactions that are mutually beneficial to self and others. In this study, we investigated the involvement of cerebellar and cortical mentalizing regions when predicting action sequences resulting from lower-level mental states, specifically intentions. Consistent with our primary cerebellar hypothesis that the posterior cerebellum would be preferentially recruited for sequencing future social actions, we did find cerebellar activation in Crus 1 that confirmed its role in sequencing (e.g., Social Sequencing > Social Selection-only contrast). However, we failed to find support for its social specificity (e.g., non significant Social Sequencing > Non-social Sequencing contrast). Interestingly, activation was found in the cerebellar lobule IX across all social conditions regardless of sequencing (e.g., all social > non-social contrasts), although there was some preference for social sequencing as well (e.g., Social Sequencing > Social Selection-only contrast). This finding is in line with the previous trait prediction study (Haihambo et al., 2021). As hypothesized, we also found activation in cortical mentalizing areas (mPFC, TPJ, and precuneus) during intention prediction, regardless of sequencing (e.g., all social > non-social contrasts). This confirms that these cortical mentalizing regions are sensitive to mentalizing but not to sequencing.

### Role of the cerebellar crus in intention-based sequence prediction

Our hypothesis that the posterior cerebellum is preferentially recruited when processing a combination of sequencing and social mentalizing information was only partly confirmed. As mentioned earlier, Crus 1 was preferentially activated during sequential prediction, but not specifically more during human as opposed to inanimate sequence prediction. One potential explanation for this lack of social specificity could be that the cerebellar Crus is involved more when confronted with high-level social information that is more abstract, such as traits, and not so much during intentions, which reflect lower-level social information that is less abstract (Van Overwalle et al., 2014). This explanation is corroborated by our previous study using the same paradigm, which revealed cerebellar Crus activation during social compared with non-social sequence prediction based on trait inferences (Haihambo et al., 2021). Other studies that focused on goal-directed behaviors, however, did find Crus activation (Li et al., 2021), although they did not focus on prediction. It also is possible that social specificity is harder to demonstrate for low-level intentions, and due to a lack of power, activations may have remained below significance levels in the present study. Note, however, that we used a comparable number of trials and participants as in our high-level trait study (Haihambo et al., 2021) with 27 participants and 44 trials (i.e., 11 trials per condition). This is similar to other intention-based mentalizing studies; for example, Atique et al. (2011) had 22 participants and 40 trials across four conditions (i.e., 10 trials per condition) and Walter et al. (2004) had 13 participants and 44 trials across four conditions (i.e., 11 trials per condition). Another potential explanation is that inanimate objects may still be perceived as somewhat goal-directed, when the description invited interpretations of self-propelledness, a common goal endpoint and action-effect of the object (Biro & Leslie, 2007), although to our knowledge, these effects were only demonstrated for visual movements of objects, not for verbal descriptions. Future studies could further investigate potential differences in specialization of mentalizing sequencing areas within the cerebellum, for instance, by directly comparing mentalizing inferences varying in high versus low abstraction level (i.e., traits vs. intentions).

It could be argued that stronger cerebellar Crus activation in sequencing versus non-sequencing is due to task difficulty. To investigate this, we computed a main Sequencing > Selection-only contrast, regardless of social or non-social domain (e.g., “Sequencing > Selection-only”; Table 4; Fig. 3). We found that posterior cerebellar mentalizing areas were no longer activated. Instead, we found robust activations in more anterior cerebellar areas in lobule IV and Crus 2. Previous cerebellar parcellations (Buckner et al., 2011; King et al., 2019) have attributed these areas to sensory motor and executive functions. While the sequencing task surely was more difficult with an additional instruction to look for appropriate sequences rather than simply reading them, this reasoning does not explain why difficulty as such would recruit mentalizing areas of the posterior Crus during social sequencing, rather than executive control areas located more anteriorly as can be seen in the sequencing main effect (Table 4). Analogously, difficulty is a confound that is almost impossible to avoid empirically for distinct types of mentalizing, because lower-level inferences that remain close to observed actions are by their very nature easier than high-level inferences, which are most distant from social input. Nonetheless, several meta-analyses have demonstrated that inferences on the state of mind of others and the self tend to recruit a limited set of areas in the mentalizing network (Schurz et al., 2014; Van Overwalle, 2009).

Considering the traditional understanding that the cerebellum is involved in motor processes, it is important to establish that our results are not a reflection of motor processes, especially because there are some motor elements in our study, such as pressing buttons to provide the chronological order of the sentences in the sequencing task. However, recall that we included a non-social counterpart which also required button presses to provide a chronological sequence. So, the same motor elements are present in both the social and non-social conditions. To investigate this, we compared Non-social Sequencing versus Non-social Selection-only conditions and Sequencing versus Selection-only main effects and found no posterior cerebellar activations for either comparison. Together, these results rule out motor processes as being responsible for posterior cerebellar activations in the Social Sequencing conditions. These results are in line with the contemporary understanding of posterior cerebellar involvement in social processes (Buckner et al., 2011; Van Overwalle et al., 2015; Van Overwalle, Ma and Heleven, 2020a).

Nonetheless, given that participants responded slower, but more accurately on social trials compared with non-social trials, it is possible that participants paid more attention to social stimuli than non-social ones. This bias in favor of social stimuli is however difficult to eliminate as social information is more salient and relevant to participants as social beings.

### Role of the cerebellar lobule IX in social sequence prediction

As noted earlier, we found consistent activation in the cerebellar lobule IX across social conditions, with a preference for Social Sequencing (e.g., Social Sequencing > Social Selection-only; Table 1; Social Sequencing > all other conditions; Table 4), as hypothesized. This was not observed in any of the non-social conditions. Beyond its inclusion in the mentalizing network during resting state (Buckner, Krienen, Castellanos, Diaz, and Yeo, 2011; Habas et al., 2009) and in nonmotor processes (Guell, Schmahmann, Gabrieli, and Ghosh, 2018), the function and role of cerebellar lobule IX during mentalizing is not well understood or documented. Perhaps the position of the cerebellar lobule IX at the inferior location of the posterior cerebellum may have resulted in it being overlooked or excluded from some standard analyses, potentially resulting in false negatives (Baetens, Firouzi, Van Overwalle, and Deroost, 2020; Habas et al., 2009). Nonetheless, evidence exists linking the cerebellar lobule IX to future event memory elaboration (Addis, Pan, Vu, Laiser, and Schacter, 2009; Addis, Wong, and Schacter, 2007) and predicting social action sequences (Haihambo et al., 2021). In the clinical realm, decreased grey matter in lobule IX and Crus 1 has been associated with autism (Cauda et al., 2011; Duerden, Mak-Fan, Taylor, and Roberts, 2012; Nickl-Jockschat et al., 2012; Stoodley, 2014; Yu, Cheung, Chua, and McAlonan, 2011), a disorder characterized by impaired social processing and limited anticipation of future social events.

This prior evidence and the position of lobule IX closer to the anterior part of the cerebellum may point to a more action-driven function, which could explain why this lobule is activated under all social predictive demands, regardless of whether a prediction involves a detailed sequence of social actions or only some rough direction or goal for an action to start (Van Overwalle, Baetens, Mariën, and Vandekerckhove, 2015). This is in line with the notion that the evolutionary older function of the anterior cerebellum is the forward control of action, implemented by constructing forward models, which aid in future planning of motor processes (Molinari et al., 1997; Ito, 2008). Although the activation observed in lobule IX mostly involved the mentalizing network, it also involved the limbic network in the Social Sequencing as opposed to Social Selection-only (MNI coordinates 20, −42, −36; Buckner et al., 2011). This finding could further support the motivational role of cerebellar lobule IX in predicting and engaging in future social sequences. To further understand the sensitivity of lobule IX to social sequences and elucidate its functions in social cognition, future studies could investigate varying levels of social abstraction during future prediction as noted above.

When comparing social processing during sequencing versus non-sequencing (Selection-only) tasks, activations were observed in attentional, executive control and somatomotor areas of Crus 1, VI, and VII, but not in mentalizing areas (Buckner et al., 2011). Considering that both conditions in the comparison included social information (i.e., Social Sequencing vs. Social Selection-only), these results may reflect a cerebellar function specific to social sequencing, further supporting the sequencing hypothesis of the cerebellum in all types of action (Van Overwalle, Manto, et al., 2020b). Notably, however is that cerebellar activation in this contrast was only observed in the left hemisphere. This unilateral activation also was observed in our previous study (Haihambo et al., 2021) for the same contrast. While there is some evidence of contralateral connectivity between the left cerebellum and right cerebral mentalizing areas (Metoki et al., 2021), these studies have focused primarily on mentalizing and not specifically on the sequencing function of the cerebellum. Further studies could investigate whether there are functional differences relating to social sequencing in the left and right cerebellum.

Recall that participants were always presented with the sequencing task before the Selection-only task. Although this presentation avoids spill-over effects, it may introduce task-order confounds. Futures studies could further investigate this.

### Past and future intentions: role of the mentalizing cortex

As hypothesized, we also found activation in cortical mentalizing areas, such the mPFC, TPJ, and PCC, which were coactivated alongside cerebellar lobule IX in the social compared with non-social contrasts, but not in sequencing compared with the non-sequencing (Social Selection-only) tasks. These activations highlight the sensitivity of these areas to social processes, rather than being specific to sequencing. Indeed, the cerebral cortex is generally less sensitive to sequencing, as opposed to the cerebellum, which has the main function of identifying temporal sequences, and to encode these into internal models to predict and perform future actions smoothly and automatically. According to Caligiore et al. (2019), computations of cortical mentalizing areas mainly involve pattern recognition, whereas cerebellar processes involve trial and error learning and prediction of these patterns. Several researchers have postulated that this error-based learning focuses on the prediction of dynamic sequences of movements and mental representations (Ito, 2008; Leggio and Molinari, 2015). Applied to mentalizing and intention prediction in the present study, this suggests that input from cerebellar internal models are transmitted to cortical mentalizing areas so that we can explicitly imagine and predict what others might do in the future. In line with this, several meta-analyses previously suggested that there is a functional overlap between mentalizing areas and autobiographical recall in the cerebrum (Andrews-Hanna, Saxe, and Yarkoni, 2014a; Spreng, Mar, and Kim, 2009) and in the cerebellum (Van Overwalle et al., 2014). Our results are further supported by other studies that found these cortical areas activated when thinking about others’ mental states (see meta-analyses by Schurz et al., 2014; Van Overwalle, 2009), as well as when imagining future intentions (Den Ouden, Frith, Frith, and Blakemore, 2005; Vogeley et al., 2001), attributing intentions to others (cartoons in social interactions; Atique et al., 2011; Brunet et al., 2000), and social intentions (Ciaramidaro et al., 2007; Walter et al., 2004).

Interestingly, the caudate was activated in all sequencing versus non-sequencing contrasts, confirming that the basal ganglia (which the caudate is a part of) may play a general role in sequencing processes, especially in motor sequencing (Baetens et al., 2020; Janacsek et al., 2020). While this area was not systematically activated in other studies on social sequencing, this may again point to a specific aspect of predicting social action, as this more closely related to future planning of motor processes than the identification of sequences in social action.

## Conclusions

The present study investigated the role of the posterior cerebellum in social action prediction based on a person’s intentions. These findings support results from our previous study on trait-based social action predictions (Haihambo et al., 2021) and extends this for the first time to predict future action sequences on the basis of intentions. These findings highlight the sequencing hypothesis of the cerebellum not only for motor and cognitive processes (Leggio and Molinari, 2015), but also for social action (Van Overwalle, Manto, et al., 2020b). Our results also point to specialized roles within the mentalizing areas of the cerebellum, in particular, the role of cerebellar lobule IX in predicting future social action sequences based on intentions, which was not documented in earlier sequencing studies involving traits (Pu et al., 2020, 2021), goal-directed trajectories (Li et al., 2021), and false beliefs (Heleven et al., 2019).

## Data Availability

All requested (pseudonymized or anonymous) data are available upon request, excluding data that allow identifying individual participants. If relevant, all manuals and code for processing the data also are available together with the data.
